# Impact of Sleep Deprivation on Emotional Regulation and the Immune System of Healthcare Workers as a Risk Factor for COVID 19: Practical Recommendations From a Task Force of the Latin American Association of Sleep Psychology

**DOI:** 10.3389/fpsyg.2021.564227

**Published:** 2021-05-20

**Authors:** Katie Moraes de Almondes, Hernán Andrés Marín Agudelo, Ulises Jiménez-Correa

**Affiliations:** ^1^AMBSONO Sleep Clinic, Department of Psychology and Postgraduate Program in Psychobiology, Federal University of Rio Grande do Norte, Natal, Brazil; ^2^Research Department of the Neurology Center, Institute of Behavioral Sleep Medicine, Medellín, Colombia; ^3^Sleep Disorders Clinic, Research Division, Medicine Faculty, National Autonomous University of Mexico, Mexico City, Mexico

**Keywords:** sleep, emotional regulation, immune system, cognitive behavioral therapy, health professionals

## Abstract

Healthcare workers who are on the front line of coronavirus disease 2019 (COVID-19) and are also undergoing shift schedules face long work hours with few pauses, experience desynchronization of their circadian rhythm, and an imbalance between work hours effort and reward in saving lives, resulting in an impact on work capacity, aggravated by the lack of personal protective equipment (PPE), few resources and precarious infrastructure, and fear of contracting the virus and contaminating family members. Some consequences are sleep deprivation, chronic insomnia, stress-related sleep disorders, and post-traumatic stress disorder. These sleep alterations critically affect mental health, precipitating or perpetuating anxiety, stress, and depression, resulting in the inability to regulate positive and negative emotions. Pre-existing sleep disorders are an important risk factor for the development and maintenance of PSTD when individuals are exposed to an important stressor such as a COVID-19 pandemic. At the same time, how an individual regulates the emotion associated with worries during daytime functioning impacts nighttime sleep, precipitating and perpetuating difficulties in sleeping. All of these changes in sleep and emotional regulation also alter the immune system. Sleep deprivation is commonly associated with chronic inflammatory diseases, due to the desynchronizations in circadian rhythms, causing possible psychophysiological disorders and impaired neuroimmune-endocrine homeostasis. From this perspective, we clarify in this article how sleep disorders affect the immune system and emotional regulation, explaining their phenomenological and neurobiological mechanisms, and discussing elements of cognitive and behavioral coping for health professionals to adopt and manage a healthier sleep pattern in the COVID-19 outbreak.

## Introduction

The current severe acute respiratory syndrome coronavirus 2 (SARS-CoV-2), the causative agent of the ongoing coronavirus disease 2019 (COVID-19) (Tay et al., 2020), has provoked a strong emotional reaction, which affects healthcare workers, symptomatic patients, and the general population. Healthcare workers who are on the COVID-19 front- line undergo shift schedules, have long and strenuous work hours with few breaks, experience circadian rhythm desynchronization, and an imbalance between effort of hours at work and reward in saving lives, resulting in an impact on work capacity, aggravated by the lack of individual protection equipment, fear of contracting the virus, or returning home and contaminating family members. Sleep disorders have been reported as one of the negative results (Belingheri et al., 2020a; Zhang et al., 2020).

The literature has reported how immune function decreases after affective states associated with stress in the face of stressful situations, such as natural disasters, among which depression, anxiety, and loneliness stand out (Ironson et al., 1997). These emotional states and the relationship with the immune response have been described and also associated with sleep disorders such as insomnia and drowsiness, as a result of sleep deprivation, establishing the important role of sleep in emotional regulation and its relationship with immune regulation (Brand et al., 2014; Irwin and Opp, 2017; Vandekerckhove and Wang, 2017).

Both emotional responses and sleep disturbances may be related to the current COVID 19 pandemic, where isolation measures, in addition to the workload, affect health professionals. Due to the burden of stress generated, sleep deprivation, little contact with their family, long hours, and concern for the future, healthcare workers could experience a decrease in their immune response and a lower response to future outbreaks in this sector of the population where COVID 19 has already claimed many victims (Alnofaiey et al., 2020; Conroy et al., 2021). These situations have already been explored in other types of populations (Dubey et al., 2020; Liem et al., 2020; Rajkumar, 2020; Rashidi Fakari and Simbar, 2020; Yang et al., 2020; Yao et al., 2020; Zhu et al., 2020).

Returning to how studies have addressed the relationship between sleep and emotional regulation in the immune system, it has been described as alterations in mitogenic responses, a decrease in the activity of NK cells and a phenotypic decrease in T cells, and the impact of catecholamines through j3 receptors on lymphocytes through the action of hypothalamic adrenal cortisol products (Ironson et al., 1997). What has not yet been analyzed is what occurs when peaks in cases rise, which has been happening in some countries despite vaccination. These peaks cause a return to isolation measures and other precautions, which causes a stabilization of symptoms of stress and sleep disorders in health professionals, which will be reflected in the decrease in the immune response, for which the measures for both prevention against stress and sleep become valid and of vital importance (Lin and Chen, 2021).

For decades, the biopsychosocial impact of sleep disorders and sleep deprivation caused by shift work schedules has been discussed (Härmä, 1993; James et al., 2017; Cheng and Drake, 2018; Kerkhof, 2018). Much research has provided evidence of intervention possibilities, but the lack of appreciation of sleep complaints by managers persists, seen by the lack of public policies for sleep disorders. Paradoxically, the COVID-19 pandemic highlighted the importance of health professionals to face this situation, but it made visible the lack of care for them.

There is consensus on how to deal with sleep disorders by major organizations (World Sleep Society, European Sleep Research Society, Sleep Brazilian Society, and the Latin American Federation of Sleep Societies) (Sateia et al., 2017; Bacelar and Pinto, 2019; Altena et al., 2020; Federation Latin-American of Sleep Societies, 2020). Cognitive-Behavioral Interventions (such as sleep hygiene) have been elected as the technical gold standard non-pharmacological treatment for many sleep disorders and sleep deprivation. Through this formal recognition, the Latin American Association of Sleep Psychology (LASP) was organized as an association that brings together sleep psychologists in Latin America with goals that involve the identification of psychological factors contributing to the development and/or maintenance of sleep disorders, contributing information to establish the differential diagnosis, in the development and provision of evidence-based cognitive-behavioral assessment and intervention techniques, collaborating to prevent sleep disorders and promoting quality of life.

In this sense, the LASP is aware of its role in the current pandemic and has formulated recommendations for the sleep complaints of the population at different ages and different social contexts, considering cultural differences between the countries of this portion of the American continent. Regarding health professionals, LASP has been concerned with the high number of sleep disorders that are characterized as insomnia in China and the informal reports, although there are no data on sleep disorders from every country. Therefore, this article aims to discuss the impact of sleep disorders on emotional regulation and the immune system. It also aims to adequately characterize these sleep disorders; insomnia is not always Insomnia Disorder, but instead may be insomnia associated with acute stress or a symptom of Posttraumatic Stress Disorder (common in these pandemic situations), or a symptom of Circadian rhythm sleep-wake disorder shift work type. Recommendations for health professionals to deal with sleep disorders are listed.

### Sleep Definition

Sleep is a global state and a universal mammalian behavior with multiple levels of biological organization (Hobson and Pace-Schott, 2002).

Sleep has been defined as a reversible state, and unlike hibernation and torpor, it is not dependent on the availability of food, water, or environmental temperature (Krystal et al., 2013).

Sleep plays an active role in processes such as synaptic plasticity and memory functions, emotional regulation, metabolic function, energy balance, macromolecule biosynthesis, removal of toxic substances and metabolic waste, and prophylactic cellular maintenance. It has also been postulated that is related to adaptive inactivity; sleep can be viewed as a process of meta regulation, that is a high order of regulation which accommodates a broad range of molecular, cellular, and network processes altogether providing optimal (adaptive) wakefulness (Vyazovskiy, 2015).

In Electrophysiology, normal human sleep is defined by two states—Rapid eye movement (REM) and Non-REM (NREM) sleep—that alternate cyclically across a sleep episode. NREM includes a variably synchronous cortical electroencephalogram (including sleep spindles, K complexes, and slow waves) associated with low muscle tonus and minimal psychological activity. REM sleep EEG is desynchronized, muscles are atonic, and dreaming is typical. On the other hand, behaviorally sleep is a reversible state of perceptual disengagement from and unresponsiveness to the environment (Carskadon and Dement, 2017).

Some factors that determine sleep manifestation, such as homeostatic and circadian timing system, environmental zeitgebers, stress, genetics, psychosocial, medical, and social features (i.e., work schedule), have been described (Borbely et al., 2016; Altena et al., 2020).

### Biological Rhythms

Molecularly, the circadian rhythm of sleep involves interlocking positive and negative feedback mechanisms of circadian genes (period -per 1,2,3-; cryptochrome -cry 1 and 2- clock and Bmal 1), and their protein products in cells of the suprachiasmatic nucleus (SCN) are entrained to ambient conditions by light. Subsequently, circadian information is integrated with information of homeostatic sleep need in the nuclei of the anterior hypothalamus (Hobson and Pace-Schott, 2002).

Episodic Ultradian rhythms have been defined as periodic rhythms that last for 20 min to 6 h, such as the patterns of the electrical activity of the brain and the heart. The functional significance of ultradian events might be in optimizing biological activities mainly by synchronizing compatible processes and preventing the simultaneous activation of incompatible processes, preparing biological systems to respond to stimuli such as cell-cell communication, and interacting with circadian rhythms (Goh et al., 2019).

Unlike ultradian rhythms, sleep and wakefulness have been named “sleep-wake cycle” and are defined as the circadian (∼24-h) rhythm. Sleep regulation has been explained with the two-process model, in which it is postulated that a homeostatic process (Process S) interacts with a process controlled by the circadian pacemaker (Process C), with time-courses derived from physiological and behavioral variables (Borbely, 2009).

The interaction between the homeostasis process (depending on sleep and wake) with a process controlled by the circadian pacemaker determines salient aspects of sleep regulation. REM Slow Wave Activity (SWA) represents the principal marker of Process S during sleep; core body temperature and melatonin rhythms are markers of process C (Borbely et al., 2016).

Process S increases in intensity, and any time that sleep occurs, it is reduced; a daytime nap, for example, causes an exponential decline in process S to the degree that it may interfere with sleep initiation at the usual bedtime. Process C influences the timing of sleepiness based on the endogenous circadian clocks (CC), the SCN of the hypothalamus, primarily by activation and deactivation of the system that promotes waking (Krystal et al., 2013).

In another sense, CC are biological fundamental functions that generate self-sustained 24 h rhythms endogenously (such as sleep-wake cycle) and help tune the organism’s physiology with the predictable cyclic environment generated by the alternation of day and night; unfortunately, social factors (such as work or school schedules) have not been adapted to the sleep-wake cycle, leading to a discrepancy between internal circadian time and external social time constraints. This- discrepancy has been named Social Jet Lag (SJL) (Korman et al., 2020).

Social jet lag can be detrimental to health and sleep health in different types of employment, including healthcare workers. Ss an example, Kang et al. (2020) studied the effect of SJL on sleep quality in nurses; they concluded that overall sleep quality can increase with decreasing day-shift fatigue, decreasing SJL and increasing sleep quality during night shifts.

### Natural Day Light Versus Artificial Light at Night

Environmental cues, mainly nighttime natural darkness, are necessary for normal melatonin synthesis that is essential for biological timekeeping, sleep, and, directly or indirectly, many processes of cells, tissues, and organs. The 24 h Light/Dark cycle of nature conveys crucial temporal cues to the body’s master biological clock (the suprachiasmatic nuclei SCN of the hypothalamus and pineal gland) to achieve internal synchronization of the period and phasing of the Circadian Time Structure CTS (Smolensky et al., 2015).

On the other hand, Artificial Light at Night (ALAN) exposure can disorganize the circadian system, from the level of the molecular clocks that regulate the timing of cellular activities to the level of synchronization between our daily cycles of behavior and the solar day (Potter et al., 2016); ALAN exposure also suppresses melatonin secretion, increases sleep onset latency, and increases alertness, causing circadian misalignment which can cause negative effects on psychological, cardiovascular, and/or metabolic functions (YongMin et al., 2015; Potter et al., 2016).

Throughout the industrialized world, 24 h operations are necessary for public safety and health and are frequently economically advantageous. A subset of shift workers develops shift work disorder (SWD) triggered by circadian misalignment (ICSD-3) (American Academy of Sleep Medicine, 2014). These individuals experience significant negative health consequences and diminished quality of life as a result of shift work (Wickwire et al., 2017).

Some consequences of circadian rhythm and sleep disruption have been described (Potter et al., 2016; Seifalian and Hart, 2019; Table 1).

**TABLE 1:** Consequences of the disruption of circadian rhythm.

Disrupted glucose metabolism (reduction in insulin sensitivity, impaired TSH Secretion, and nocturnal cortisol secretion increased after sleep deprivation).
Effects on dietary choices (sleep disruption increases non-homeostatic eating propensity and accentuates increased activity in brain regions involved in reward in response to food stimuli, increased appetite, particularly for energy-dense food.
Limited daylight exposure (many individuals are sheltered from the beneficial effects of natural daytime light on behavior and physiology due to a vitamin D deficiency.
Increased light exposure at night (light exposure shortly after dusk during workdays, delaying sleep onset and shortened sleep duration; this has gotten worse due to the use of electronic devices at bedtime and during the day).
Night workers have higher plasma triacylglycerol, circadian misalignment increases blood pressure (mainly during sleep) and inflammatory markers, reverses cortisol rhythms, and reduces heart rate variability and insulin sensitivity in healthy adults.
Disfunction in the gastrointestinal and cardiovascular system are known to be a risk for colorectal and breast cancer.
Impaired cognitive performance and increased frequency of errors in those suffering from regular sleep disturbance; it includes the cognition of health care workers that are providing treatments and therapy to patients in the hospital.

## Sleep Deprivation, Emotional Regulation, and The Immune System

The immune response protects the organism from substances or organisms that are probably harmful or dangerous. There are many studies that the literature has used to argue the importance of sleep within this immune response and how sleep deprivation influences, in an important way, its regulation (Wilder-Smith et al., 2013; Irwin, 2015).

A first account of this relationship is that during sleep and through its role in the consolidation of long-term memory, such consolidation in the immunological memory is necessary and effective, which occurs during deep slow-wave sleep (stage N3 of sleep), allowing an abstraction of the immune system to remember its action against specific pathogens, in addition to other specific memory threads during the REM state of sleep, which is also related to emotional regulation, which is associated with the decrease of adrenergic loads, which favor immune action (Westermann et al., 2015). It is here where both sleep deprivation and increased responses to stress can alter this process, making the body vulnerable to pathogenic actions, even in the respiratory system, as has been shown in studies that argue that short periods of sleep deprivation are associated with susceptibility to common colds, evidenced and also related to adaptive immunity (Prather and Leung, 2016; Lin et al., 2018).

A second aspect that is important to highlight is that sleep deprivation has a close relationship with two components of our immune response. One such component is innate or non-specific immunity, referring to the defense system with which one is born that forms the first line of defense in the immune response. The other component is related to acquired, adaptive, or specific immunity. It is made up of highly specialized cells and systemic processes that eliminate or avoid the threats of pathogens, generating immune memory and tolerance to the antigens themselves (Wilder-Smith et al., 2013; Irwin, 2015).

### Sleep Deprivation and Adaptive Immunity

When analyzing the adaptive immune response and its relationship with sleep deprivation, the literature has considered that the activity of the hypothalamic-pituitary-adrenal axis is responsible for the distribution of glucocorticoid hormones through the blood serum, to regulate gene expression in practically every cell in the body. Sleep deprivation causes hormonal activation of leukocyte glucocorticoid receptors, resulting in profound suppression of antiviral gene programs (Wilder-Smith et al., 2013; Irwin, 2015).

Sleep deprivation also gives rise to activation of the sympathetic nervous system (SNS), releasing norepinephrine in primary and secondary lymphoid organs, in all other major organ systems, including our vascular and perivascular tissues, as well as many other peripheral tissues, and stimulates the adrenal glands, also releasing epinephrine. Both neuromediators stimulate leukocytes and adrenergic receptors (e.g., ADRB2) to suppress genetic antiviral (IRG) interferon response (IFN) gene programs, mediated by regulation factors of IRF interferons (Wilder-Smith et al., 2013; Irwin, 2015).

Other studies have linked sleep with the induction of growth hormone release which occurs in the early part of the night. This hormone is involved in improving the proliferation and differentiation of T cells and promoting the activity of type 1 cytokines (Wilder-Smith et al., 2013; Irwin, 2015). According to the above, sleep deprivation reduces the release of growth hormone and suppresses the response of Genetic antiviral interferon (IRG), mediated by IRF regulatory factors, which causes an imbalance in Th1 to Th2 cells, with decreased IFN production in Th1 cells and increased production of interleukin-Th2 cells. 10 (IL-10). This suppression of the adaptive immune response has been hypothesized to contribute to a greater susceptibility to infectious diseases and a lower response to vaccines (Wilder-Smith et al., 2013; Irwin, 2015).

### Sleep Deprivation Innate Immunity

After sleep deprivation, the SNS releases norepinephrine in the primary and secondary lymphoid system and stimulates the adrenal release of epinephrine (Wilder-Smith et al., 2013; Irwin, 2015). Both neuromodulators stimulate ADRB2 leukocyte adrenergic reception and activate inflammatory systems, which are mediated by nuclear factors (NF) -κB and intrinsic circuits, that in turn are responsible for the detection of microbes through pattern recognition receptors (PRR), among which is the Toll-like-4 receptor (TLR4). This stimulates inflammatory gene expression through NF-κB transcription factors and the production of proinflammatory cytokines, such as interleukin (IL) -6 and tumor necrosis factor-α (TNF-α) (Wilder-Smith et al., 2013; Irwin, 2015).

Homeostasis between the internal and external signals of the brain allow it to regulate inflammatory activity. It can also influence brain activity and alter internal balances, sleep being one of the most affected (Wilder-Smith et al., 2013; Irwin, 2015). Therefore, when sleep dysregulation occurs, it can confer an increased risk of inflammatory factors, resulting in cardiovascular disease, cancer, and emotional disorders (Tobaldini et al., 2013). That is where the relationship of the emotional system appears in this triad and at the same time these factors have been considered mortality factors for the transmission of SARS-CoV-2.

### Sleep and Emotional Regulation

Sleep deprivation also affects the regulation of emotional processing. This condition makes us more emotionally reactive and more sensitive to stressful stimuli and events. Scientific literature has shown how sleep appears to be essential to our ability to cope with emotional stress in everyday life. However, when daily stress is not properly regulated, it can lead to mental health problems and sleep disorders (Vandekerckhove and Wang, 2017). Not only does emotion impact sleep, but there is also evidence that sleep plays a key role in regulating emotion. Emotional events during waking hours affect physiology, sleep patterns, and even the content of daydreams, and the quality and quantity of sleep influence how we react to events that affect our overall well-being (Bileviciute-Ljungar and Friberg, 2020).

Different investigations have shown how sleep has a modulating action on daily emotions, specifically in the interaction between emotional stress, sleep, and its disturbances. Regular sleep from its homeostasis, circadian presence, and in the respective development of REM—NREM cycles is crucial for general well-being and emotional experience during the day (Vandekerckhove and Wang, 2017). This is observed in the first measure, because our executive functions exert a modulating action on our experience and emotional reaction, evidenced in the correct functioning of our frontal limbic connections, improving when REM sleep is intact. The correct processing of negative experiences also occur during REM sleep, which is important in the consolidation of affective memory and allows emotional stabilization in disorders such as depression (Killgore, 2010; Vandekerckhove and Wang, 2017).

Sleep deprivation alters this regulation and makes the person more reactive in the face of aversive reactions, showing a decrease in mean prefrontal activity and its signals sent to the amygdala, which translates into emotional dysregulation (Minkel et al., 2011; Saghir et al., 2018). Psychophysiological factors such as stress, anxiety, and hyperarousal play an important role in causing sleep disturbances. Furthermore, sleep disturbance predicts later development of mental health, while the development of insomnia predicts psychopathology such as depression or post-traumatic stress disorder after an acutely stressful event (Ironson et al., 1997). This could be what happens in health professionals who are exposed to sleep deprivation due to long hours of work and also face high levels of stress when they are away from their home and/or when they remain in the medical setting because of the concern of contagion generated by living with family after caring for COVID-19 patients.

**FIGURE 1:**
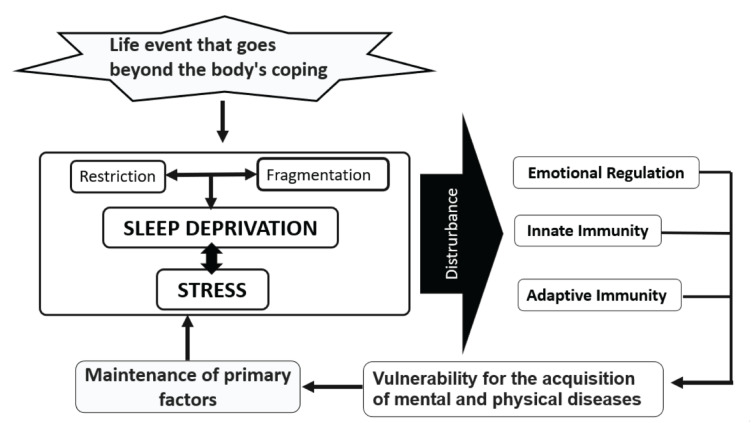
Model of vulnerability and maintenance of the disease due to sleep deprivation and stress (source authors). It can be seen how stressful events that exceed the body’s ability to respond to them generate sleep deprivation and stress; these last two factors form a relationship that occurs in a bidirectional way because one can be the cause of the other. Regarding sleep, it can be affirmed that the causal relationship of deprivation can be facilitated by voluntary and involuntary restriction of the subject or by fragmentation of the same or both factors, which cause sleep deprivation to increase. As a result of this interaction, the alteration of emotional regulation and the immune response, both acquired and adaptive, arises, which puts the subject, after the alteration of said factors, at greater vulnerability for the acquisition of mental and physical diseases, which increases the primary factors of stress and sleep deprivation, which is not linear and can create a perpetuation of these problems or the acquisition of diseases such as COVID. This demonstrates the importance of prevention measures in healthcare workers.

This leads us to conclude a bidirectional role of the action of sleep deprivation, either by restriction or fragmentation on stress in the first place and stress on sleep deprivation second. While it is true that stress causes sleep disturbances, sleep deprivation is a high source of psychological and physiological stress (Meerlo et al., 2008; Van Laethem et al., 2015). For a better understanding, the Model of vulnerability and maintenance of the disease, due to sleep deprivation and stress, is shown in Figure 1.

One of the main aspects of this regulation is the regulatory action of cortisol, a hormone involved in the control of stress and reactivity against emotions. It seems that the regulatory alteration of the action of melatonin on cortisol, which is one of circadian disturbances, explains said emotional reactivity and an alteration of the circadian cycle due to sleep deprivation or lack of sleep creates said emotional dysregulation (Posadzki et al., 2018; Brignardello-Petersen, 2019; Meng et al., 2020; Shermohammed et al., 2020).

Sleep deprivation has the consequence of inhibiting the previous processes, causing emotional reactivity and chronic stress, which has been related to chronic diseases, similar to those that occurred in the current COVID pandemic, which leads to the conclusion that the alteration in emotional regulation and circadian lag makes the population vulnerable to contracting COVID, or becoming more vulnerable to the consequences of the disease.

## Sleep Alterations in Healthcare Workers

As discussed above, the literature data converge with published data on the situation of healthcare workers during the current outbreak of COVID-19, showing that the perceived poor sleep quality and sleep changes appear to be underlying mechanisms in the relationship between shift and work overload, increased susceptibility to infection, and impact on mental health (Brooks et al., 2018; Belingheri et al., 2020b; Pappa et al., 2020).

The first study published by the research group in Wuhan, China, where the pandemic started (Zhang et al., 2020), showed in a sample of 1,563 individuals that 564 participants (34.6%) had insomnia during their work in the hospitals in Wuhan, and that related factors included isolation, psychological concerns about the outbreak of COVID-19 (uncertainty about effective disease control), and being a doctor. Complementary data from Xiao et al. (2020b) with 180 doctors and nurses who treated patients with COVID-19 infection in Wuhan, China, showed that the staff had poor sleep quality and explained that the associated factors were the energy expended for putting on Personal protective equipment (PPE) every day and the disinfection needed for removing these clothes, the continuous work the isolation wards with a high intensity of high-pressure work, and the high mortality rates of patients associated with the infection by COVID-19. These data were replicated by the population, showing high rates of poor sleep quality associated with stress and anxiety in the context of COVID-19 (Huang and Zhao, 2020).

Data have shown that health professionals complain of insomnia that causes sleep deprivation and bad sleep quality. However, it is necessary to characterize that insomnia can be a chronic condition by itself, caused by the pandemic context, or a symptom of another sleep disorder. This differential diagnosis is relevant to thinking about cognitive and behavioral recommendations for this group.

It is known that the biological disaster of COVID-19 is a stressful situation, as being infected with a life-threatening physical illness is a traumatic event. Further, being a healthcare worker for patients with a deadly virus and having prolonged contact with them can also lead to symptoms of acute stress. Sleep and post-traumatic stress disorder (PTSD) are closely related. Literature data showed that the COVID-19 pandemic appears to be a risk factor for sleep disorders and PSTD (Casagrande et al., 2020; Yin et al., 2020). Further, Richards et al. (2020) showed sleep alterations (sleep deprivation, sleep fragmentation, and insomnia) could lead to maladaptive sleep-related compensatory behaviors and cause hyperarousal and anxiety-related disorders like PTSD. Moreover, sleep disorders are core features of PTSD. Therefore, insomnia, associated with another sleep-wake disorder, mental disorder, or medical condition, can only be diagnosed as an independent focus of clinical attention, if these conditions are treated and insomnia persists.

Insomnia disorders are characterized by the complaint of persistent difficulty with sleep initiation, duration, consolidation, or quality that occurs despite adequate opportunity and circumstances for sleep. These symptoms should cause clinically significant impairment in social, occupational, educational, academic, and behavioral functioning; otherwise, they will not be classified as Insomnia Disorder. For the diagnosis of Insomnia Disorder, it is necessary to observe the duration and frequency of complaints. The situational difficulties of sleep due to negative environmental circumstances, such as during the COVID-19 pandemic, should be differentiated. Insomnia may be a symptom of Circadian rhythm sleep-wake disorders shift work type (CRSWD) (International Classification of Diseases—11th revision—ICD-11). The CRSWD are persistent or recurrent disturbances of the sleep-wake cycle due to alterations of the circadian time-keeping system, its entrainment mechanisms, or a misalignment of the endogenous circadian rhythm and the external environment (social demands, work and school schedules, or the light-dark environment). The most common complaints are excessive sleepiness or insomnia, or even both (International Classification of Diseases—11th revision—ICD-11; International classification of sleep disorders, 3rd edition.—ICSD-3) (American Academy of Sleep Medicine, 2014).

The CRSWD shift work type is associated with significantly higher odd burnout syndrome and job dissatisfaction (Bagheri Hosseinabadi et al., 2019). Furthermore, the disruption of circadian rhythm may impair immune system function, among other consequences, as mentioned previously (Cuesta et al., 2016).

## Behavioral and Cognitive Recommendations for Sleep Quality in Health Professionals During the COVID-19 Pandemic

Many protective recommendations have been put forward to deal with the SARS-CoV-2 pandemic, but no scientific society around the world (World Health Organization, 2018), including WHO, has devoted itself to sleep problems, except for t h e task force of the European CBT-I Academy (Altena et al., 2020).

Researchers from China, the initial epicenter of the pandemic, have discussed social support (size and source of social networks of people helping others, as well as emotional, material, and supportive functions informative—Brugha, 1990) and capital social (social trust, belonging, and social participation—Harpham et al., 2004) as important mediating factors to improve sleep quality (Xiao et al., 2020a) and to help in the sense of self-efficacy by professionals (that refers to individual judgment on the ability to complete a certain behavior or task—Bandura, 1977).

In this sense, it is important to consider the risk and protective factors to suggest behavioral and cognitive recommendations to sleep quality in health professionals during the COVID-19 pandemic, to help with social support, social capital, and a sense of self-efficacy. Factors that negatively affect sleep, in addition to those mentioned above (pandemic stress, work pressure, or irregular or night shift work patterns), include loneliness, negative family environment, technology use, evening light, pre-sleep worry, and the use of caffeine, tobacco, and alcohol. Fear of missing out on family contact and their health and excessive technology use negatively impact sleep. Persons with comorbid medical, psychiatric, and other sleep disorders such as sleep apnea, and individuals with a strong need for stable hours of sleep, may be at particular risk. Factors that positively affect sleep include social support, good family environments, good sleep hygiene, and physical activity (Altena et al., 2020; Federation Latin-American of Sleep Societies, 2020).

The literature maintains that behavioral and cognitive intervention strategies (Mullins et al., 2014; Aliyu et al., 2018; Marín Agudelo et al., 2019; Almondes, 2020; Altena et al., 2020; Federation Latin-American of Sleep Societies, 2020; Holding et al., 2020) can be applied as preventive measures for health personnel, in addition to the classic norms of sleep hygiene and finding preventive measures before work and operative measures during work (Table 2).

**TABLE 2:** Behavioral and Cognitive measures for health professionals dealing with sleep problems during COVID-19.

**Before work**	**Operational**
• Avoid starting work tasks with very high sleep debt. • Track total sleep time compared to hours of sleep with the most drowsiness. • Schedule short naps of maximum 30 min based on this. • Maintain, as much as possible, the same amount of sleep in a workday as in a non-workday. This standard is the one that most favors the immune response. • Assessing the use of more than one sleep period as much as possible reduces the potentially serious consequences of strenuous hours. • Taking a nap if possible when you are sleepy is the most efficient method of countering cognitive errors. • Exposure to bright light before work, particularly blue spectrum light, is alerting. • Limit napping just before work to 30 min to reduce sleep inertia problems. • In free time, try to exercise regularly, but not before bedtime or before naps at work breaks. • Encourage the choice of relaxing activities, including socializing with family, before going to sleep or napping—reading a book using a relaxation technique. • When you sleep at home during the day, educate family members or roommates about sleep and the importance of restful sleep. The family must help protect the health professional’s sleep from factors such as neighbors, pets, and delivery people. • Helping techniques also include turning the phone off, turning off the TV, or putting it on a white noise channel, wearing earplugs, darkening the bedroom, wearing eye masks, and sleeping in a cool environment. • The management of luminosity before and after work with dark glasses is necessary to collaborate with the regulation of the circadian cycle. • Food intake involves light meals, at specific times, if possible, and not immediately before the start of sleep, to avoid sleep disturbances due to digestion.	• Try to obtain natural light during the day. If it is not possible, use bright lights at work, but not in the room or place where you sleep at work. The idea is to obtain little light in the place where you sleep to help in the pressure to sleep. Suggesting the use of sleep masks that help to darken the environment to favor sleep is a good strategy. • Social interaction with preventive isolation (teamwork), chewing on snacks, singing, and physical exercise can help maintain alertness. • It is recommended to use short exercise breaks (for example, climbing stairs) of at least 6 min and to work in well-lit spaces. • Finally, avoiding excessive consumption of energy drinks, such as caffeine and stimulant self-medication, should also be considered. Doctors must remember that it is unclear whether these drugs restore executive functions after sleep deprivation and it remains unclear how long you can stay alert to compensate for a lack of sleep. • Unfortunately, some health professionals may be kept away from their family or community due to stigma or fear. This can make a situation much more difficult. If possible, suggest that the health professional remain connected with loved ones, even though digital methods, which is a way to keep in touch. Talk to colleagues or other trusted people for social support and to express stress and other emotions and concerns about the work situation during the day—colleagues may be having similar experiences. • Limit exposure time to COVID-19 news as much as possible so as not to exacerbate anxiety. • Create an outline of personal care activities that the professional likes, when not taking care of patients, such as spending time with friends (virtually) and with family, exercising, or reading a book. • Learning from signs and symptoms—paying attention and differentiating sleepiness, fatigue, fear, feelings of sadness, withdrawal, guilt, anxiety, encouraging them to seek breaks, and asking for professional help. This includes psychological help. • If the health workers identify symptoms related to sleep deprivation, fatigue, errors in performing work-related tasks, inability to concentrate or make decisions, extreme irritability, or strong emotional reactions, should inform colleagues and superiors and take a nap. Even a short nap can help partially reduce these symptoms.

## Final Considerations

In order to improve decision-making and reduce the risk of errors at work, it is urgent that the work protocols, with patients with COVID 19, contain measures to improve the sleep of health personnel.

It is important that the recommendations mentioned above are used to reinforce a positive appraisal of the situation with the help of sleep psychologists, avoiding the development of psychopathological conditions and sleep disorders, in order to deal with this situation. It is important to encourage positive coping styles. Coping represents the cognitive and behavioral patterns to manage particular external and/or internal demands appraised as taxing or even exceeding the resources of individuals (Folkman and Lazarus, 1988). In our opinion, three different coping strategies will help the physical and mental health of professionals, since practical behaviors such as emphasizing positive cognitions, understanding sleep alterations and emotional regulation, and getting more information can be associated with fewer mental health problems and sleep problems (Dubey et al., 2020; Guo et al., 2020; Wang et al., 2020): (1) Coping focused on evaluation involves attempts to understand sleep alterations and the cognitive and behavioral variables involved in the context of the COVID-19 pandemic, with information and professional support; (2) Coping focused on problem involves the development of a coping plan, seeking internal resources to find solutions to deal with the situation, and redefining thoughts to be more positive; (3) Coping focused on emotions involves individual control of emotions and emotional balance, involving efforts to maintain hope when dealing with a stressful situation through emotional regulation, psychoeducation and sleep hygiene techniques, becoming aware, and engaging in pleasurable activities that bring a sense of accomplishment.

Finally, for future post-pandemic phases, it is important to formulate public policies for decisions and actions in the face of sleep disorders.

## Conclusion

The occupational field of the health professional during the pandemic brings with it an increase in the workload and a displacement of sleep schedules, causing sleep deprivation and increased stress. Both stress and its deprivation have a bidirectional relationship, intimately linked to the immune system and the regulation of emotions, which creates an increase or presence of sleep disturbances, emotional disturbances, and the appearance of immunological vulnerability.

The literature has reported that these aspects can be prevented through strategies that must be carried out before and after work, in order to mitigate the aforementioned problems and establish better coping strategies both for the COVID 19 pandemic and for problems and contingencies that may arise in the future. The main objective when preparing this document was to present concrete tools that have served in other similar situations and apply them at this time for health professionals. A further aim was to ensure that in future perspectives, faced with similar problems, these tools can be the starting point to improve the quality of life of health professional in times of these crises, which is why this working group meets and this approach is presented for health professionals.

For future consensus and working groups, it remains for us to return to these issues raised and conduct research that will allow us to affirm these recommendations objectively.

## Author Contributions

KA, HA, and UJ-C: study design, writing the draft, integration of the authors’ comments, and final manuscript. All authors contributed to the article and approved the submitted version.

## Conflict of Interest

The authors declare that the research was conducted in the absence of any commercial or financial relationships that could be construed as a potential conflict of interest. The reviewer RK declared a past collaboration with one of the authors KA.
